# Comment on ‘Domestic light at night and breast cancer risk: a prospective analysis of 105000 UK women in the Generations Study’

**DOI:** 10.1038/s41416-018-0203-x

**Published:** 2018-12-25

**Authors:** Christopher Conrad Maximillian Kyba, Manuel Spitschan

**Affiliations:** 1 GFZ German Research Centre for Geosciences, Potsdam, Germany; 2 Leibniz Institute for Freshwater Ecology and Inland Fisheries, Berlin, Germany; 3 Department of Experimental Psychology, University of Oxford, Oxford, UK

**Keywords:** Breast cancer, Physics

We read with great interest the recently published article “Domestic light at night and breast cancer risk: a prospective analysis of 105000 UK women in the Generations Study” by Johns et al.^1^ The study examined the relationship between breast cancer risk and self-reported levels of artificial light in the bedroom, and the 95% confidence levels of the hazard ratios that were consistent with little or no effect. In this letter, we would like to draw attention to a major problem with the instrument used for the subjective estimation of light exposure, and to suggest a different instrument for future studies.

The four levels of exposure were: “light enough to read”; “light enough to see across the room, but not read”; “light enough to see your hand in front of you, but not to see across the room”; and “too dark to see your hand, or you wear a mask”. We propose that the two middle levels have no meaningful difference, and that it was therefore incorrect for Johns et al. to assign “light enough to see across the room, but not read” together with “light enough to read” into a so-called “high” light at night (LAN) exposure.

Broadly speaking, “to see” refers to the ability to encode sufficient contrast to differentiate between different objects or surfaces. From first principles, there are three conditions in which it is possible to see your hand in front of you: first, both your hand and the background could be illuminated at levels sufficient for the human visual system to operate. We believe that this is likely to be the case in the vast majority of urban bedrooms. Second, your hand could be illuminated, and seen against the contrast of a black background. Third, you could observe your hand as a black shadow against an illuminated background. The second and third possibilities would require quite contrived lighting design, so in nearly all cases if you can see your hand, you can also see across the room.

**Fig. 1:**
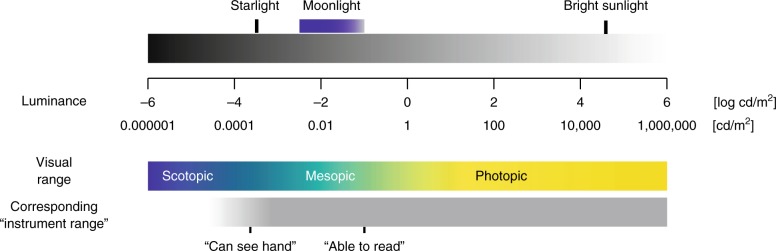
Light levels and approximate visual ability. Luminance values are based on reflection from a nearly white surface. Both “light enough to see hand” and “light enough to see across the room” are possible starting at or near starlight levels, and thus provide equivalent information about the luminance levels in typical bedrooms. Note that exact visual performance levels depend on many factors, including previous light exposure, pupil size, and local contrast

When light enters a bedroom, it will reflect off objects, the walls, the ceiling, and the floor. While there will surely be differences in illuminance between these surfaces (depending on their intrinsic properties such as their bidirectional reflectance distribution function), if there is light, then there is light everywhere. People with normal visual ability are easily able to orient under starlight^2^ (0.6–0.9 mlux), which is many orders of magnitude less than the illuminance required to read (Fig. 1). At starlit levels, it is certainly possible to see the walls and objects in a room, once the eye has had a few minutes to dark adapt.

We have personally noted that people often misjudge their visual abilities when recalling past experiences. Urban dwellers not used to allowing their eyes time to dark adapt will often incorrectly claim that a rural area was “so dark you couldn’t see your hand in front of you”. Similarly, under light levels of around 0.1 lux, one can still see so well that it is a disquieting experience to realise that reading small text is not possible.

We would like to propose an alternate set of questions for self-assessment: “sleep with mask or blackout curtains” (Dark); “cannot walk through bedroom without turning on a light” (Dark); “can see around room, but not enough light to read” (Dim); “a bright light shines through the window” (Dim); “a light source (lamp or TV) is left on during the night” (Light). We suspect that asking about concrete objects or behavior is likely to better correlate with actual light levels.

We realise, however, that there are fundamental challenges to asking participants to estimate their light exposure, even using a methodology clamped to specific natural tasks such as the above. These challenges include significant temporal delay between the time the survey is given to the participants, and the time the survey is asking about, and thus distortion by memory. At the same time, a subjective instrument is necessary to estimate light exposure in a large population, simply for practical reasons. The validity of any instrument must therefore be evaluated by actually measuring the light in bedrooms of a representative subset of the participants after they have completed the questionnaire.
